# Safety Assessment and Pain Relief Properties of Saffron from Taliouine Region (Morocco)

**DOI:** 10.3390/molecules27103339

**Published:** 2022-05-23

**Authors:** Maroua Ait Tastift, Rachida Makbal, Thouria Bourhim, Zineb Omari, Hiroko Isoda, Chemseddoha Gadhi

**Affiliations:** 1 Laboratory of Agri-Food, Biotechnology, and Valorization of Plant Resources, Phytochemistry and Pharmacology of Medicinal Plants Unit, Faculty of Sciences Semlalia, Cadi Ayyad University, Avenue Prince Moulay Abdellah, BP 2390, Marrakesh 40000, Morocco; marouatastift@gmail.com (M.A.T.); makbal.rachida@gmail.com (R.M.); t.bourhim@gmail.com (T.B.); zineb.omari4@gmail.com (Z.O.); 2 Agrobiotechnology and Bioengineering Center, CNRST-Labeled Research Unit (AgroBiotech-URL-CNRST-05 Center), Cadi Ayyad University, Marrakesh 40000, Morocco; 3 Faculty of Life and Environmental Sciences, University of Tsukuba, Tennodai 1-1-1, Tsukuba City 305-8572, Ibaraki, Japan; isoda.hiroko.ga@u.tsukuba.ac.jp; 4 Alliance for Research on the Mediterranean and North Africa (ARENA), University of Tsukuba, Tennodai 1-1-1, Tsukuba City 305-8572, Ibaraki, Japan

**Keywords:** *Crocus sativus*, stigma, analgesia, receptor systems, Moroccan saffron

## Abstract

Saffron is the most expensive spice in the world. In addition to its culinary utilization, this spice is used for medicinal purposes such as in pain management. In this study, the analgesic activity of *Crocus sativus* stigma extract (CSSE) was evaluated in rodents and its possible physiological mechanism was elucidated. The anti-nociceptive effect of CSSE was evaluated using three animal models (hot plate, writhing, and formalin tests). The analgesic pathways involved were assessed using various analgesia-mediating receptors antagonists. The oral administration of CSSE, up to 2000 mg/kg, caused no death or changes in the behavior or in the hematological and biochemical blood parameters of treated animals nor in the histological architecture of the animals’ livers and kidneys. CSSE showed a central, dose-dependent, anti-nociceptive effect in response to thermal stimuli; and a peripheral analgesic effect in the test of contortions induced by acetic acid. The dual (central and peripheral) analgesic effect was confirmed by the formalin test. The anti-nociceptive activity of CSSE was totally or partially reversed by the co-administration of receptor antagonists, naloxone, atropine, haloperidol, yohimbine, and glibenclamide. CSSE influenced signal processing, by the modulation of the opioidergic, adrenergic, and muscarinic systems at the peripheral and central levels; and by regulation of the dopaminergic system and control of the opening of the ATP-sensitive K^+^ channels at the spinal level. The obtained data point to a multimodal mechanism of action for CSSE: An anti-inflammatory effect and a modulation, through different physiological pathways, of the electrical signal generated by the nociceptors. Further clinical trials are required to endorse the potential utilization of Moroccan saffron as a natural painkiller.

## 1. Introduction

Pain is a physiological process whose purpose is to warn the person of a threat to his physical integrity, hence the term nociception [1]. However, to this simple transmission of the nociceptive message from the periphery to the somato-sensitive cortical centers is added an emotional and behavioral component, which explain the complex and multifactorial nature of pain.

Suffering from pain, especially chronic pain, has become a major medical and socio-economic burden worldwide. Pain reduces the quality of life and has physical, psychological, and environmental impacts not only on the individual but also on society as a whole [2].

The intervention of central and peripheral mechanisms in pain in the majority of cases makes the choice of an adequate pharmacological therapy very difficult. The analgesics that act at the central level, in spite of their great effectiveness, are not dissociated from side effects, namely addiction, constipation, and respiratory depression [3,4]. Furthermore, analgesics that proceed at the peripheral level have undesirable effects, such as gastrointestinal and renal lesions [5,6]. In this sense, several studieshave been devoted to discover new natural resources with analgesic potential and low secondary effects.

Saffron, also known as *Crocus sativus* L. stigmas (Iridaceae), is the most expensive spice in the world. At the retail price, it costs 4000 to 6000 US dollar per kg of dried stigmas and requires the collection of 200,000 to 300,000 flowers for one kg of saffron. The flowers are manually handled to gently remove the three precious stigmas that make up saffron. The major saffron-growing countries around the world are Iran, India, Morocco, Spain, Greece, Afghanistan, and Italy [7,8,9,10]. Iran produces 90% of the saffron that exists on the market, making it the leading producer and exporter in the world.

Morocco occupies an important place in the saffron market [11]. The country is the world’s fourth largest producer of this expensive spice, with an annual production of 6.5 tons in 2019, grown over an area of 1865 Ha, mainly in the region of Taliouine in the Anti-Atlas Mountains [12].

The chemical composition of saffron has been widely studied [8,10,13]. Stigmas mainly contain secondary metabolites belonging to the terpenoid family, in particular monoterpenes and apocarotenoids, and to the flavonoid family, all glycosidic derivatives of kaempferol [8,11,14,15,16].

Among the apocarotenoids, crocetin and its glycoside esters, crocins, are the most important [8] while safranal, a monoterpene aldehyde, and its glucoside derivative, picrocrocin, are the other two most important phytochemicals abundant in *C. sativus* stigmas.

The quality of saffron depends on its coloring potential, its taste, and its aroma, ascribable to crocin, picrocrocin, and safranal contents, respectively. Three categories of saffron are defined according to the saffron ISO-norm 3632-2: 2010 [17], namely category 1 (the highest quality category), category 2 (intermediate), and category 3 (lowest quality).

Although saffron samples from different locations or countries have similar overall chemical composition, it has been reported that there are qualitative and/or quantitative differences in chemical composition between samples; differences that are related to the geographical origin, drying process, and storage conditions [8,11,14,18,19,20,21]. Thus, it impacts the organoleptic properties and beneficial health effects of the saffron sample. The differences concern the structure of crocin (the glycoside ester of crocetin), the composition of the volatile fraction, and the structure of the kaempferol derivatives [8,14,20,21].

In saffron samples, different crocin derivatives exist depending on the isomer (cis/trans) of crocetin involved and on the number of glucose molecules attached to the molecule of crocetin [8,20,21]. Kaempferol derivatives also differ from one sample of saffron to another depending on the structure of sugars moiety [8]. Nevertheless, the key difference concerns the aromatic volatile fraction. Indeed, in addition to the drying process [11,18,20], the geographical location affects the chemical profile of the volatile fraction of saffron [14].

Karabagias et al. [14] compared saffron from Greece, Spain, Iran, and Morocco, on the basis of volatile compounds. They reported significant variations in the volatile compounds content, according to the geographical origin of the saffron, finding the following pattern: Morocco > Iran > Greece > Spain. The GC/MS analysis revealed that in all samples, safranal was the most abundant volatile compound but its relative content differs depending on saffron samples. Safranal represented 79.04, 71.35, 76.42, and 64.71% of saffron’s total volatile fraction, in Moroccan, Greek, Iranian, and Spanish samples, respectively [14]. Regarding other volatile safranal derivatives, a significant difference was recorded among samples in relation to the geographical origin [14].

Saffron has been used as a spice, dye, and for medicinal purposes for nearly four millennia [7]. It is considered safe in therapeutic doses [15,22]. Different studies reported the non-toxic effects of saffron after acute oral administration of a dose of 2 g/kg [15,22] and even at doses up to 5 g/kg [8,23]. No symptoms of toxicity were recorded after repeated administration of low doses of saffron (28 days of administration of a dose of 100 mg/kg in the rat) [22] while repeated oral administration of higher doses (>5 g/kg) was likely to cause vomiting, uterine contractions, dizziness, etc. [24].

Various health-promoting properties have been accredited to it and its main metabolites, crocin, crocetin, picrocrocin, and safranal, namely anticancer, anti-Alzheimer’s, anti-Parkinson’s, improved memory and learning abilities, treatment of anxiety disorders and schizophrenia, anti-nociceptive, anti-inflammatory, hepatoprotective, antidiabetic, macular degeneration protection, anticoagulant, skin anti-aging, skin lightening, antisolar, and moisturizing properties [9,13,15,16,19,25,26,27,28,29,30,31,32,33].

In recent times, saffron has gained a special interest for its antidepressant properties based on in vivo testing and randomized controlled trials [34,35,36,37,38]. The inhibition of serotonin reuptake has been proposed as a possible mechanism of action of saffron and crocin [34]. It has also been acclaimed for its anti-cancer potential [39,40,41,42,43,44]. In a very recent report, El Midaoui et al. (2022) [45] highlighted the role of saffron in the treatment of neuropsychiatric and age-related diseases. Saffron and its major compounds have powerful antioxidant and anti-inflammatory properties in vitro and in vivo [46,47]; properties that would be partly responsible for the effectiveness of saffron and its metabolites in the treatment of diseases [44,48,49,50].

Saffron is traditionally known to ease pain. Its analgesic activity was evaluated in vivo and in clinical trials [15,30,31,32,33]. The anti-nociceptive effect was found by Hosseinzadeh and Younesi [30] to not be significant in mice and to not involve the central nervous system, while other studies have confirmed saffron’s effectiveness [31,32,33], and the involvement of central mechanisms [15], without, however, specifying which ones are involved.

The majority of studies have used, in their investigation, saffron from Iran for its convenience, Iran being the first producer and exporter of this spice in the world [24] However, despite Morocco’s place on the podium of saffron production, and even with the strong aroma that characterizes it (high yield in volatile fraction [14]), Moroccan saffron has not been investigated for its functionality and, therefore, could hide unexpected assets.

Thus, the aim of the present work was primarily to assess, for the first time, the analgesic properties of saffron from Morocco. Furthermore, the elucidation of the physiological mechanism involved in the analgesic activity of *C. sativus* stigma extract (CSSE) using various antagonists of analgesia-mediating receptors was also recorded.

## 2. Results

### 2.1. CSSE Had No Toxic Effect

The acute toxicity was studied using the limit dose test reported in the Food and Drug Administration (FDA) and OECD guidelines [51,52]. The limit dose of 2000 mg/kg body weight is the recommended dose for acute toxicity studies to avoid the unnecessary use and killing of animals (one of the pillars of animals handling).

**Figure 1:**
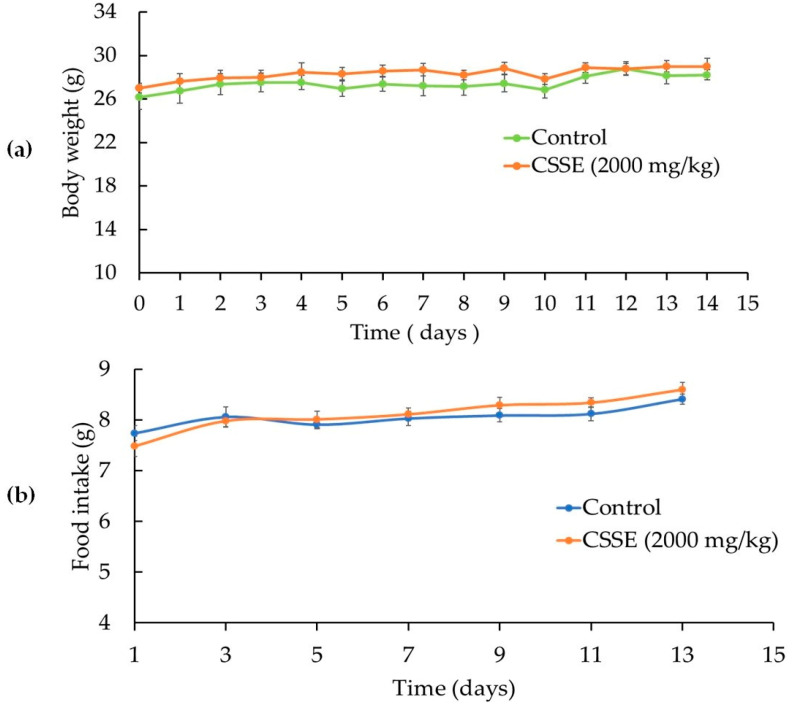
Evolution of the body weight (**a**) and the food intake (**b**) of mice treated with a single dose of CSSE (2000 mg/kg, p.o.). CSSE: *C. sativus* stigma extract. The values are expressed as mean ± SEM (*n* = 6). No significant difference was recorded between CSSE-treated group and non-treated control (*p* < 0.05, ANOVA I Tukey’s test).

**Figure 2:**
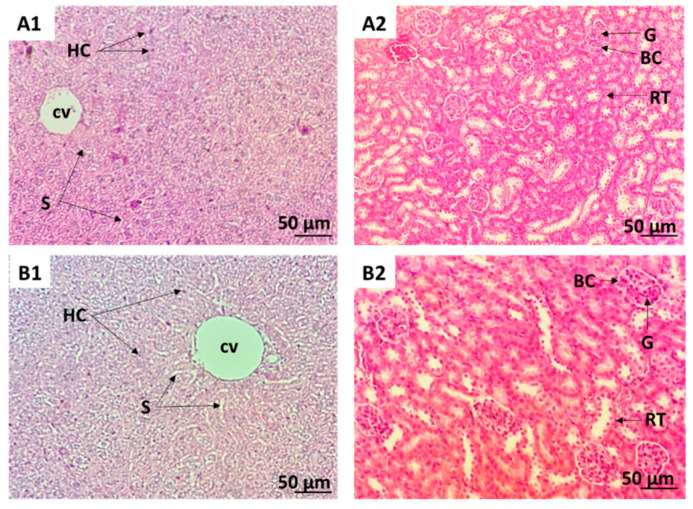
Photomicrograph (hematoxylin-eosin staining; ×200 magnification) of liver and kidney sections of mice after administration or not of a single oral dose of CSSE (2000 mg/kg). (**A1**): liver section of control mice. (**B1**): liver section of mice treated with CSSE. (**A2**): kidney section of control mice, (**B2**): kidney section of mice treated with CSSE. CV: Central vein, HC: Hepatocytes, S: Sinusoids. G: Glomerulus, BC: Bowman’s capsule, RT: Renal tubule. Analyses of liver and kidney sections showed no significant histopathological changes after a single oral administration of CSSE (2000 mg/kg) compared to the control group (*n* = 6 per group).

The oral administration of a single dose of CSSE (2000 mg/kg body weight) to mice did not cause any death within the fourteen days of the study. No toxicity symptoms or adverse behavioral changes were observed in CSSE treated mice (Table 1). In addition, the extract did not affect mice body weight (Figure 1a) nor the amount of food intake (Figure 1b) compared to the control. No macroscopic alterations were observed in the mice’s organs during the autopsy and the relative organ weights were unaffected (Table 2). Hematological parameters showed no significant difference between the treated mice and the control group (Table 3). Biochemical analysis of serum aspartate aminotransferase (AST) and alanine aminotransferase (ALT) showed that treatment with CSSE did not cause significant changes in serum transaminases (Table 3). In the same way, no changes in creatinine, urea, alkaline phosphatase, and total bilirubin levels were observed compared to the control group. The data show that the biochemical parameters remained within the physiological range, indicating that CSSE did not affect liver and kidney function. Histopathological analysis of the liver and kidneys was performed and confirmed the macroscopic examination (Figure 2). The oral median lethal dose (LD50) of CSSE was much higher than 2000 mg/kg body weight of mice. Therefore, CSSE was considered as safe according to the FDA [51] and the OECD [52] guidelines.

### 2.2. Analgesic Activity

#### 2.2.1. CSSE Reduced the Abdominal Contractions Induced by Intraperitoneal Injection of Acetic Acid in Mice

Acetic acid is typically used as noxious stimuli in visceral pain studies [53]. When injected intraperitoneally to mice, it induces abdominal writhing (stretching, retracting, or pressing the belly against the floor). The writhing test is based on counting these events and is used to assess the pain threshold for inflammatory agents [53].

The pretreatment with CSSE significantly decreased the writhing response in a dose-dependent manner with a high inhibition rate of writhing (71.6%) observed at 200 mg/kg; while at 50 mg/kg and 100 mg/kg, the inhibition rates induced by acetic acid were of 48.3% and 60.5%, respectively. This pain-relieving effect of CSSE was very significant, as shown by its relative activity when compared to morphine (1 mg/kg, i.p). At 200 mg/kg, CSSE exhibited an 84.7 % morphine equivalent inhibitory effect (Table 4).

#### 2.2.2. CSSE Increased Mice Latency Time to Respond to Heat-Induced Pain

**Figure 3:**
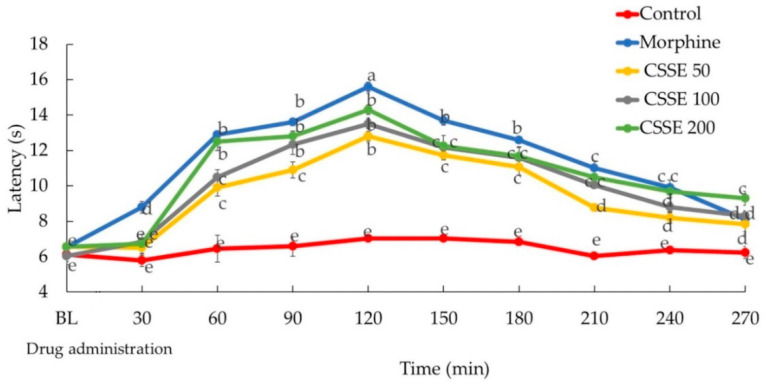
Effect of a single administration of CSSE (50, 100, or 200 mg/kg) on the latency time of mice to react to heat in the hot plate test. Morphine (1 mg/kg, i.p) is used as positive control. Each value represents the mean ± SEM (*n* = 6); different letters indicate a significant difference during the duration of the experiment or between treatments (*p* < 0.05) (ANOVA II Duncan’s test).

Hot plate test is used to evaluate the pain threshold of mice towards heat [54]. CSSE showed a significant analgesic effect in the hot-plate test from 60 min after the start of treatment. Mice which received CSSE showed a significant, time-dependent increase in latency time to react to heat in comparison with the control group. The maximal analgesic effect in CSSE-treated group was observed at 120 min, as for morphine. After 4 h from the start of treatment, the pain threshold of mice towards heat decreased. (Figure 3).

#### 2.2.3. CSSE Inhibited the Pain Induced by Formalin Injection in the Animal Paw

The formalin test is used to differentiate between acute and chronic pain [55]. The animal reacts to the pain triggered by the chemical stimuli by licking its paw. Paw licking time was recorded for the first five minutes after the formalin injection as an indicator of acute pain and between the 15th and 30th min of injection, indicating chronic pain [56].

**Figure 4:**
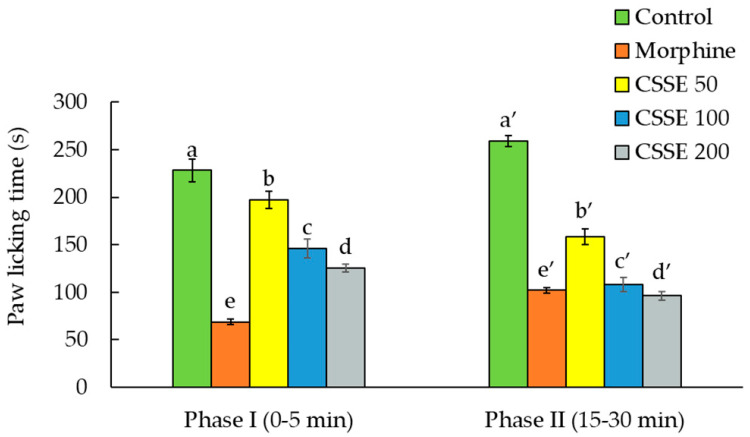
Effect of a single administration of CSSE (50, 100, or 200 mg/kg, p.o.) on paw licking time of mice treated with formalin. Morphine (1 mg/ kg, i.p) is used as positive control. Each value represents the mean ± SEM (*n* = 6); different letters (a–e,a′–e′) indicate a significant difference between treatments (*p* < 0.05) (ANOVA I Duncan’s test).

The pain induced by the injection of the formalin solution in the mice paw was significantly inhibited by CSSE and morphine in both phases of the formalin test, the early phase and the late phase. CSSE (50, 100, or 200 mg/kg, p.o.) significantly reduced the time spent by mice licking their paws after formalin injection, during the early phase, by 13.4%, 35.9%, and 45%, respectively, and during the late phase by 38.8%, 53.6%, and 62.8%, respectively (Figure 4). The anti-nociceptive effect of CSSE was more noticeable in the late phase than in the early phase (Figure 4); while morphine was more effective in the early phase. Morphine significantly inhibited pain by 69.8%.

### 2.3. Mechanistic Studies of CSSE Anti-Nociceptive Effect

Nociceptive transmission involves several mediators acting through several pathways. The possible anti-nociceptive mechanisms of CSSE were investigated by pre-treatment of mice with different antagonists in the writhing test and the hot plate test.

#### 2.3.1. Involvement of the Opioidergic Pathway in CSSE Analgesic Effect

To evaluate the involvement of opioid receptors, mice were pretreated with an opioid receptors’ antagonist, the naloxone (1 mg/kg, i.p.) [57].

**Figure 5:**
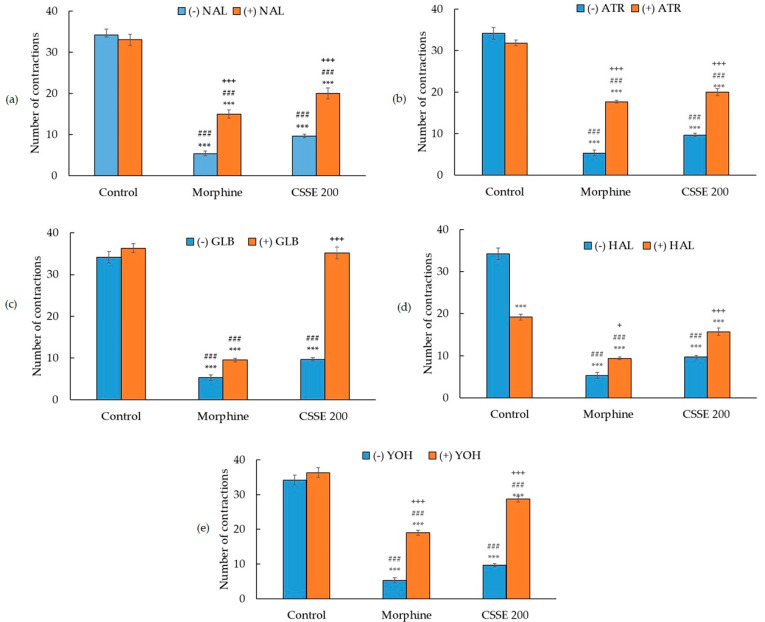
Effect of CSSE on the nociceptive response to abdominal contractions induced by injection of acetic acid in mice pretreated with or without naloxone (**a**), atropine (**b**), glibenclamide (**c**), haloperidol (**d**), and yohimbine (**e**); 15 min before administration of CSSE or morphine. Each value represents the mean ± SEM (*n* = 6); Statistical significance was calculated by ANOVA I followed by Tukey’s test. *** *p* < 0.001 when comparing all groups to the control group; ### *p* < 0.001 when comparing all groups to the control group pretreated with naloxone (**a**), atropine (**b**), glibenclamide (**c**), haloperidol (**d**), and yohimbine (**e**); + *p* < 0.05, +++ *p* < 0.001 when comparing CSSE-treated group or morphine-treated group with the corresponding group pretreated with naloxone (**a**), atropine (**b**), glibenclamide (**c**), haloperidol (**d**), and yohimbine (**e**).

In the writhing test, naloxone significantly (*p* < 0.001) reversed the effect of both CSSE and morphine by increasing the mean number of abdominal contractions (Figure 5a). The analgesic effect of CSSE and morphine decreased in the presence of naloxone by 42% and 33.6%, respectively.

**Figure 6:**
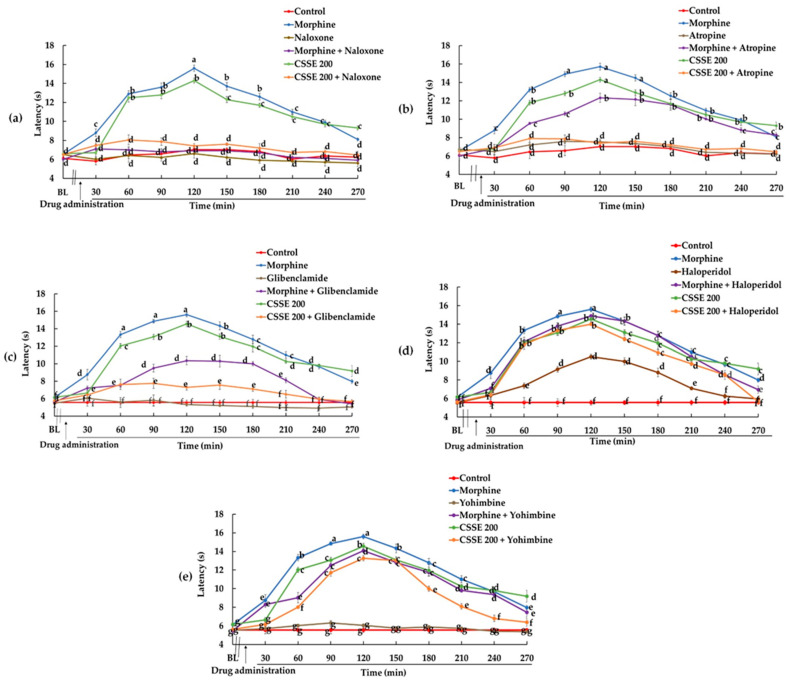
Effect of CSSE on the latency time of mice to react to heat in the hot plate. Mice were pretreated with or without naloxone (**a**), atropine (**b**), glibenclamide (**c**), haloperidol (**d**), and yohimbine I 15 min before administration of CSSE or morphine (**e**). BL: Base Line. Each value represents the mean ± SEM (*n* = 6); different letters (a–g) indicate a significant difference during the duration of the experiment or between treatments (*p* < 0.05) (ANOVA II Duncan’s test).

In the hot plate model, naloxone lowered the pain threshold of CSSE-treated mice to equal that of the control group as for morphine (Figure 6a).

#### 2.3.2. Involvement of the Cholinergic Pathway

To evaluate the involvement of the cholinergic receptors, mice were pretreated with a muscarinic acetylcholine receptors (mAChRs) antagonist, the atropine (1 mg/kg, i.p.) [58].

In the writhing test, atropine significantly (*p* < 0.001) increased the number of abdominal writhes of mice pretreated with morphine or CSSE (Figure 5b). Atropine reduced CSSE’s anti-nociceptive effect by 42%.

In the hot plate test, atropine significantly (*p* < 0.05) reversed the analgesic effect of CSSE back to baseline; while the analgesic effect of morphine was partially reversed (Figure 6b).

#### 2.3.3. Involvement of ATP Channel Blocker Pathway in CSSE Analgesic Effect

Another pathway potentially involved in pain generation is the depolarization of the cells. Glibenclamide is an inhibitor of ATP-sensitive K^+^ channels [59,60,61].

In the writhing test, the administration of glibenclamide (10 mg/kg, p.o.) to CSSE-pretreated mice totally abolished CSSE’s analgesic effect. The mean number of abdominal writhes produced by acetic acid increased by 100% in this group to be similar to the control (Figure 5c). Meanwhile, glibenclamide did not significantly change the mean number of abdominal writhes of mice treated with morphine. Morphine’s analgesic effect reduced only by 14.5% in the presence of glibenclamide (Figure 5c). In the hot plate test, glibenclamide partially reversed the analgesic effect of morphine and CSSE (Figure 6c).

#### 2.3.4. Involvement of Dopaminergic Pathway in CSSE Analgesic Effect

The active mechanism of haloperidol is to block the postsynaptic dopamine (D2) receptors [62]. It was therefore used to assess if CSSE acts through this pathway.

In the writhing test, the analgesic effect of morphine and CSSE decreased in the presence of haloperidol by only 14% and 24.4%, respectively (Figure 5d). In the hot plate test, haloperidol did not reverse the analgesic effect of CSSE and only slightly reversed that of morphine (Figure 6d). In contrast, haloperidol alone exerted a significant analgesic effect compared to the control with a pain inhibitory activity of 43.9% (Figure 6d).

#### 2.3.5. Involvement of the Adrenergic Pathway in the Mode of Action of CSSE

Yohimbine blocks presynaptic alpha-2 adrenergic receptors [63].

Yohimbine significantly (*p* < 0.001) increased the mean number of acetic acid-induced writhing in groups pretreated with CSSE or morphine. The analgesic effect of CSSE was greatly reduced (77%) in the presence of yohimbine as well as for morphine (47.5%) (Figure 5e). In the hot plate test, yohimbine very slightly lowered the pain threshold of mice treated with CSSE or morphine (Figure 6e).

### 2.4. Chemical Characterization of Moroccan Saffron According to ISO 3632 Guidelines

The quality of saffron is standardized by the norm for saffron ISO 3632 2:2010 [17]. The characterization of saffron samples was assessed by measuring the amount of picrocrocin (flavor strength), safranal (aroma strength), and crocin (coloring strength), and by the moisture content (expressed as percentage). The results (Table 5) showed a high content of crocin (5.6 times the required minimal limit for the first quality category), a high content of picrocrocin (7.5 times the minimal limit for the first quality category), and a high content of safranal, which ranges into the limits established by the norms for the first quality category (min. 20; max. 50). The moisture content (10.3 ± 0.24%) was lower than the maximum limit required for all categories (12%). Therefore, and according to the limits established by the ISO 3632 2:2010 [17], this saffron is classified in the first category, which refers to saffron of a high quality.

## 3. Discussion

Saffron is a spice traditionally known for its health benefits [16]. It is considered safe in therapeutic doses [15,22] but becomes toxic in high doses [24]. In the present study, the safety of Moroccan saffron was evaluated in order to further assess its analgesic activity at doses without toxic symptoms. CSSE has been shown to be safe up to a dose of 2 g/kg. No death or signs of toxicity were recorded in the treated group, which is consistent with the literature reports on saffron [15,22]. Therefore, it was possible to perform pharmacological tests in the range of safe doses and to assess the pain-relieving potential of CSSE.

The anti-nociceptive activity of CSSE was evaluated in this study using three tests: the hot plate test, writhing test, and formalin test.

The writhing test corresponds to the counting of abdominal stretching induced by the injection of acetic acid into the peritoneal cavity of mice [53]. Acetic acid, a chemical irritant, induces inflammation and is responsible for the release of chemical mediators such as serotonin, histamine, bradykinin, substance P, and prostaglandins (PGE2α) [64,65,66]. These chemical mediators stimulate peripheral chemo-sensitive nociceptors, induce an increase in vascular permeability [65], and result in abdominal writhing [66]. Therefore, because the pretreatment with CSSE significantly decreased the writhing response in a dose-dependent manner, CSSE most likely has an anti-inflammatory effect whereby it modulates nociception. This suggestion was backed up by the results of the formalin test. CSSE significantly reduced, in a dose-dependent manner, the time mice spent licking their paws after formalin injection.

The injection of the formalin solution into the mouse’ paw causes a biphasic response [55,67]. The early neurogenic pain phase is initiated immediately after formalin injection and is characterized by stimulation of the C fibers and release of the substance P and bradykinin. The second phase is due to the local inflammatory pain caused by the production of serotonin, histamine, and prostaglandins [68,69]. The results revealed that CSSE has acted effectively in both phases of the formalin test with a more noticeable effect in the late inflammatory phase than in the early neurogenic pain phase (Figure 4). Thus, CSSE acts largely in the management of pain through the control of inflammation.

Several studies have indeed confirmed the anti-inflammatory activity of saffron [15,28,45,46] and ascribed it to the activity of its metabolites, in particular crocetin, the active form of crocin. Crocetin acted through its modulatory effect on redox balance [46], regulatory activity on inducible nitric synthase (iNOS) expression, and through the inhibition of cyclooxygenase-1 and cyclooxygenase-2, and prostaglandins production [45].

CSSE also controls pain through central neurogenic pain management, as confirmed, in addition to the formalin test, by the hot plate test. The hot plate test is a central model that has a selectivity for opioid-derived analgesics [70].

CSSE showed a time-dependent anti-nociceptive effect by increasing the pain threshold of mice towards heat. This result was similar to that reported by Khan et al. [15] but does not corroborate the conclusions of Hosseinzadeh and Younesi [30], who reported that the aqueous-ethanolic and aqueous extracts of a saffron from Iran have no significant effects on latency time, hence no central analgesic effect. This discrepancy may be related to the difference in the chemical composition, which in turn is related to the geographical and environmental conditions [14].

To understand how CSSE modulates the electric signal triggered by the nociceptors, it is essential to specify which endogenous pathways have been involved and at which level (spinal and/or supraspinal). To answer the question of the level of signal integration and control, the acetic acid-induced writhing test was used to confirm the spinal control level and the hot plate test for the supraspinal one [54,71]. As the signal integration mechanism involves the release of various neurotransmitters, receptor antagonists of these neurotransmitters were used to verify their involvement in CSSE-induced analgesia.

Naloxone (non-selective opioid receptors antagonist [57]), atropine (non-selective muscarinic receptor antagonist [58]), glibenclamide (ATP-sensitive K^+^ channels blocker [59,60,61]), haloperidol (dopamine receptors antagonist [62]), and yohimbine (a selective α2-adrenoceptor antagonist [63]) were used to substantiate the involvement of the opioidergic, cholinergic, ATP-sensitive K^+^ channels, and dopaminergic and adrenergic systems, respectively.

Naloxone totally reversed the analgesic effect of CSSE in the hot plate test and partially reversed it in the writhing test. This presumes the involvement of opioid receptors [72] in the analgesic effects of CSSE, mainly at the supraspinal level, but also at spinal level, even in the peripheral endings of the primary afferent fibers, because opioids µ-receptors are also located in the periphery [72]. A similar scheme was found with atropine, a muscarinic receptor antagonist. This suggests that CSSE may also involve the cholinergic pathway in its analgesic activity at the supraspinal and spinal levels. In particular, this is because the activation of the muscarinic receptors in the dorsal horn of the spinal cord has been reported to contribute to the analgesic effect by releasing inhibitory interneurons, reducing nociceptive transmission [73].

The implication of the adrenergic system in the analgesic activity of CSSE was confirmed as well. Yohimbine, a selective α2-adrenoceptor antagonist, helped to reduce the analgesic activity of CSSE, mainly at the spinal level, with a 77% decrease in analgesia in the acetic acid-induced writhing test. The supraspinal level of action of CSSE on the adrenergic system was moderate, as confirmed by the slight decrease in CSSE activity in the hot plate test in the presence of yohimbine. These findings corroborate what was reported concerning the involvement of the adrenergic system in nociception at the spinal and supraspinal levels, mediated through activation of α- adrenoceptors and descending inhibitory pathways [74,75]. They also corroborate what was reported concerning the stimulatory effect of saffron’s metabolite crocetin on ß2-adrenoceptors [76], which contribute to crocetin antinociception activity.

Among the neurotransmitters, the dopaminergic system also plays a role in pain control [77]. Dopamine receptors are expressed in primary nociceptors as well as in the spinal neurons located in different laminae of the dorsal horn of the spinal cord, suggesting that dopamine may modulate pain signals by acting on both presynaptic and postsynaptic targets [78]. Decreased levels of dopamine have been associated with painful symptoms [77]. At the physiological level, haloperidol reduces dopamine availability by blocking the dopamine D2 receptors [79]. Haloperidol reduced CSSE-induced antinociception in the writhing test, which suggests that CSSE modulated the dopamine system at the spinal level through activation of the D2 receptors.

At the supraspinal level, haloperidol did not modify the central anti-nociceptive effect of CSSE (Figure 6d), which implies that CSSE analgesic activity in the brain certainly did not involve D2 receptors, although it may still act on the dopamine system but through other mechanisms. This assumption was supported by previous reports on saffron’s effect on the brain [80,81]. It has been reported that brain dopamine concentration is increased by saffron [80]. In line with this report, Monchaux De Oliveira et al. [81] recently established that the saffron-induced improvement of depressive-like behavior was associated with the modulation of monoaminergic neurotransmission, in particular changes in serotonergic and dopaminergic neurotransmission. Saffron modulated the dopamine neurotransmission in the frontal cortex by reducing its catabolism and in the striatum by significantly increasing local dopamine levels [81].

The generation of many pain signals in the human nervous system is mediated by ion channels [79]. Open ATP-sensitive potassium channels produce analgesia by reducing neuronal excitability and by inhibiting the release of various neurotransmitters in the spinal cord [79]. Glibenclamide, a sulfonylurea, is an oral hypoglycemic drug that acts through the inhibition of the ATP-sensitive K^+^ channels in the pancreatic cells, which leads to the depolarization of the cells and insulin secretion [82].

At the spinal level, in the writhing test, the analgesic effect of CSSE was fully reversed by glibenclamide, suggesting that the pain-relieving activity of CSSE was certainly due to the opening of the ATP-sensitive K^+^ channels. At the supraspinal level, CSSE increased the pain threshold, but to a lesser extent, through the opening of the K^+^ channels, because the glibenclamide only partially reversed the analgesic effect of CSSE in the hot plate test.

Over all, the mechanism behind the analgesic effect of CSSE is multimodal. CSSE reduced nociception, through its anti-inflammatory action, demonstrated by the formalin test and by the modulation of the electrical signal generated by the nociceptors. CSSE influenced signal processing, both peripherally and centrally, by the modulation of the opioidergic, adrenergic, and muscarinic systems; and at the spinal level, by the modulation of the dopaminergic system and the opening of the ATP-sensitive K^+^ channels.

## 4. Materials and Methods

### 4.1. Plant Material

The stigmas of *C. sativus* were purchased in November 2018 from local producers in Sktana, Taliouine region (South of Morocco). The stigmas were authenticated by Prof. A. Ouhammou, a taxonomist at Cadi Ayyad University. A voucher specimen (MARK11120) has been deposited in the regional herbarium MARK, Faculty of Sciences Semlalia, University Cadi Ayyad, Marrakech, Morocco.

### 4.2. Standards and Reagents

Acetic acid and ethanol were purchased from VWR International (Rosny-sous-Bois, France). Formalin, atropine, naloxone, and urethane were purchased from Sigma-Aldrich (St. Louis, MO, USA). Glibenclamide was bought from Promopharma (Had Soualem, Morocco). Haloperidol was procured from Pharma5 (Casablanca, Morocco). Yohimbine was purchased from Micro Ingredients (Montclair, CA, USA). Morphine was provided by Sothema (Casablanca, Morocco). Hematoxylin and eosin were purchased from Sigma-Aldrich (Merck KGaA, Darmstadt, Germany).

### 4.3. Animals

The in vivo study was carried out using adult Swiss albino mice (12 weeks) housed in cages under standard conditions (25 ± 2 °C, 12/12 h light/dark cycle) with free access to water and food. The animals were divided into groups with 6 mice per group. They were acclimatized for one week prior to the experiment and fasted overnight before each test. All studies were authorized by the Ethical Committee for Animal Care of the Faculty of Sciences Semlalia, University Cadi Ayyad, Marrakech, Morocco, in accordance with the European decree, related to the ethical evaluation and authorization of projects using animals for experimental procedures, 1 February 2013, NOR: AGRG1238767A. All mice were handled by the 3R principles of laboratory animal care and use.

### 4.4. Preparation of CSSE Extract

Saffron stigmas were grounded to powder just prior to extract preparation. Stigmas powder (0.5 g) was extracted by 5 mL of 70% ethanol, with mechanical stirring (150 Hz/min) for 48 h at room temperature and in the dark. The extract was then centrifuged for 10 min at 3000 rpm/min. A second extraction was carried out on the marc by macerating them for 48 h in 5 mL of 70% ethanol. After centrifugation, the same operation was repeated with maceration times of an additional 48 h in order to exhaust the plant material. The supernatants from the various extractions were combined and then evaporated to dryness under reduced pressure at 45 °C.

### 4.5. Acute Toxicity Test

The acute toxicity was studied by limit dose test [51,52]. Albino mice were divided into two groups (*n* = 6 per group). Control group received distilled water and test group was treated orally with CSSE (2000 mg/kg in H_2_O). Observation of the behavior of the animals was carried out every 30 min for 4 h on the first day and once a day for 14 days in order to record any signs or symptoms of intoxication, namely the modifications of the autonomous activity, piloerection, respiratory rhythm, presence of hemorrhage, diarrhea, and death. Food intake and body weight were also recorded daily. At the end of the observation period, animals were anesthetized with urethane (1 g/kg, i.p.). Blood was collected in a tube and centrifuged at 3000 rpm at 4 °C for 10 min to obtain the serum for biochemical analysis. Animals were then sacrificed, and the organs were removed, weighed, and stored in the formaldehyde solution for histopathological analysis [51,52]. Biochemical analysis of serum samples was performed using an automatic chemistry analyzer (Cobas Integra 400 plus analyzer, Roche). The biochemical parameters measured were alanine aminotransferase (ALT), aspartate aminotransferase (AST), creatinine, urea, alkaline phosphatase (ALP), and total bilirubin. Hematological analysis was performed using an automatic hematological analyzer (ABX MICROS 60-OT). The hematological parameters analyzed were white blood cell count (WBC), red blood cell count (RBC), platelets (PLT), red cell distribution width (RDW), hemoglobin (HGB), hematocrit (Hct), mean corpuscular volume (MCV), mean corpuscular hemoglobin (MCH), mean corpuscular hemoglobin concentration (MCHC), red cell distribution width (RDW), mean platelet volume (MPV), neutrophile, lymphocyte, and monocyte.

Finally, the acute toxicity of CSSE was evaluated by the determination of the median lethal dose (LD50), the evaluation of signs of intoxication at behavioral and biological levels, and the target organs.

The relative organ weight of each animal was calculated as follows:

relative organ weight = absolute organ weight (g) × (body weight of mice on sacrifice day (g))^−1^ × 100.

Liver and kidney samples from each treatment group were subjected to histopathologic examination. Organs were fixed in 10% formalin, then tissues were dehydrated and embedded in paraffin blocks. Sections of 4 µm thickness were stained with hematoxylin-eosin (H&E). Then, the slides were examined under a light microscope at 200× magnification. 

### 4.6. Analgesic Activity

#### 4.6.1. Acetic Acid-Induced Writhing Test

The analgesic activity of CSSE was deduced by the decrease in the frequency of writhing induced by acetic acid injection [53]. Mice (*n* = 6/group) were treated with 10 mL/kg, b.w, (p.o.) of distillated water (control), 50, 100, or 200 mg/kg (p.o.) of CSSE, or 1 mg/mL (i.p.) of morphine. The choice of these doses was based on the literature review. After 30 min of administration of the extracts, the animals were intraperitoneally injected with 0.6% of acetic acid. Then, the number of writhes produced in the mice within 15 min was counted for 20 min [56]. The analgesic effect was measured by calculating the reduction in the mean number of abdominal writhing for each group as compared to the control. The inhibition rate was calculated by applying the following formula:

Inhibition rate = 100 × (mean number of writhes of control group—mean number of writhes of drug treatment group) × (mean number of writhes of control group)^−1^.

#### 4.6.2. Hot Plate Test

The analgesic activity of CSSE was tested by hot plate test, as described by Laughlin et al. [83]. Swiss albino mice were divided into 5 groups (*n* = 6 per group). Group (I) was assigned as a control and received 10 mL/kg, b.w of distillated water. Groups (II, III, and IV) were administered CSSE orally at doses 50, 100, and 200 mg/kg, respectively. Group (V) was given i.p. 1 mg/mL of morphine. The hot plate was maintained at 55 ± 1 °C for a maximum time of 15 s to prevent the animals from being burnt. The latency time (time for which mouse remains on the hot plate (55 ± 0.1 °C) without licking or flicking of hind limb or jumping) in seconds was determined before and after the administration of the extract every 30 min for 4 h.

#### 4.6.3. Formalin-Induced Paw-Licking Test

The formalin-induced nociceptive behavior was performed as described by Kim et al. [84]. Mice were divided into 5 groups (*n* = 6 per group). Three groups orally received CSSE at different doses (50, 100, or 200 mg/kg); one group assigned as positive group received morphine (1 mg/kg, i.p.), and the control group was administered distilled water (10 mL/kg, p.o.). After 30 min of oral administration of the test substances, 20 μL of 1% formalin in saline solution was injected subcutaneously into the hind paw of the mouse. The animal was immediately placed in an observation cage for 30 min. The nociceptive response was assessed by quantifying the licking time of the paw during the early phase (0–5 min) and the late phase (20–30 min) after injection.

### 4.7. Mechanistic Studies

Nociceptive transmission involves several mediators acting through several pathways. The possible anti-nociceptive mechanisms of *C. sativus* stigma were investigated by pre-treatment of mice with different antagonists in the writhing test and the hot plate test [85]. The doses of antagonists were chosen based on the previous literature data. The dose (200 mg/kg) for CSSE was chosen on the basis of its high analgesic efficacy.

#### 4.7.1. The Examination of the Effect of CSSE on Opioidergic System

The possible involvement of CSSE analgesic action on opioidergic pathway was investigated using the hot plate test and the writhing test. Seventy-two mice were divided into groups with six animals each. The first 6 groups were separated as follow; groups 1 and 2 received an oral administration of distilled water (10 mL/kg, b.w) and CSSE (200 mg/kg b.w), respectively. In parallel, groups 3 and 4 were injected by morphine (1 mg/kg, i.p) and naloxone (1 mg/kg, i.p, a non-selective opioid receptor antagonist [57]). Meanwhile, groups 5 and 6, for which naloxone was administrated 15 min earlier, were treated with morphine (1 mg/kg, i.p) and CSSE (200 mg/kg, p.o.), respectively. After 30 min, the hot plate test was performed. The same treatment groups were used with the remaining six groups for the torsion test.

#### 4.7.2. The Inspection of CSSE Nociceptive Activity on Cholinergic System

To inspect the possible analgesic action of CSSE on the cholinergic system, 12 groups of mice were divided so that they had 6 mice per group, and then the mice were used for the hot plate and writhing test. The first 6 groups were divided as follow: the groups 1, 2, and 3 received distilled water (10 mL/kg, p.o.), CSSE (200 mg/kg, p.o.), and morphine (1 mg/kg, i.p), respectively. The atropine (5 mg/kg, ip, a muscarinic receptor antagonist [58]) was pre-injected to groups 4, 5, and 6. After 15 min, groups 4, 5, and 6 received distilled water (10 mL/kg, p.o.), morphine (1 mg/kg, i.p), and CSSE (200 mg/kg, p.o.), respectively. After 30 min of treatment, the hot plate test was carried out for all mice. The same treatment groups were used with the remaining six groups for writhing test.

#### 4.7.3. The Investigation of the CSSE Effect on K^+^-ATP Channel Blocker Pathway

The mechanism action of CSSE on the ATP-sensitive potassium channel blockers system was examined by acetic acid-induced writhing test. In this study, the glibenclamide was used as a K^+^ ATP channel blocker [59,60,61]. Twelve groups with six mice each were treated. The groups 1, 2, and 3 received distilled water (10 mL/kg, p.o.), CSSE (200 mg/kg, p.o.), and morphine (1 mg/kg, i.p), respectively. The groups 4, 5, and 6 were injected with glibenclamide (10 mg/kg, p.o.) 15 min prior to the administration of distilled water (10 mL/kg, p.o.), morphine (1 mg/kg, i.p), and CSSE (200 mg/kg, p.o.), respectively. After 1 h from the previous treatments, acetic acid-induced writhing test was proceeded for all groups. For another 6 groups of 6 mice each, the same treatments were applied and the hot plate test carried out.

#### 4.7.4. The Study of CSSE Analgesic Effect on Dopaminergic Pathway

To elucidate the possible contribution of CSSE on dopaminergic pathway, haloperidol was used as a dopamine receptor antagonist [62]. For this purpose, 12 groups were used. The groups 1, 2, 3, and 4 were given distilled water (10 mL/kg, p.o.), CSSE (200 mg/kg, p.o.), morphine (1 mg/kg, p.i), and haloperidol (1 mg/kg, p.o.), respectively. The groups 5 and 6 were pre-treated with haloperidol (1 mg/kg, p.o.) 15 min prior to the administration of CSSE (200 mg/kg, p.o.) and morphine (1mg/kg, i.p), respectively. The acetic acid-induced writhing test was realized for the first six groups of animals after 1 h of mentioned treatments and the others were used for the hot plate test with the same treatments

#### 4.7.5. The Evaluation of CSSE Analgesic Effect on Adrenergic System

To assess the potential involvement of CSSE on the adrenergic system, yohimbine was used as an α2 adrenergic antagonist [63]. A total of 12 groups of animals (6 mice each) were used for the writhing and hot plate test. The groups 1, 2, 3, and 4 were administrated with distilled water (10 mL/kg, p.o.), CSSE (200 mg/kg, p.o.), morphine (1 mg/kg, i.p), and yohimbine (1 mg/kg, i.p), respectively. The groups 5 and 6 were pre-injected 15 min earlier with yohimbine (1 mg/kg, ip), then they received CSSE (200 mg/kg, p.o.) and morphine (1 mg/kg, i.p), respectively. After 1 h, all animals were subjected to acetic acid-induced writhing test. The other groups were treated with the same treatments in the hot plate test.

### 4.8. Chemical Characterization of Moroccan Saffron According to ISO 3632 Guidelines

The norm ISO 3632-2 for saffron [17] describes methods suitable for testing the spice saffron, which is obtained from the flowers of the saffron crocus (*C. sativus* L.). It refers to the method for the determination of the main characteristics of saffron (picocrocine, safranal, and crocine) by spectrometric method.

Three repetitions of 0.1 g of stigma powder were macerated in 200 mL of distilled water with mechanical stirring (150 Hz/min) for 1 h in the dark at room temperature. A total of 10 mL of the solution was poured into a 100 mL flask, adding distilled water until sorted. After dilution, the solution was filtered. The absorbance of the various components of saffron was measured by a spectrophotometer UV-VIS at the following wavelengths; 257 nm (picrocrocin), 330 nm (safranal), and 440 nm (crocin). The results were expressed according to the following formula:

A1% 1 cm (λmax) = (D × 10,000)/(m × (100 − wMV)(1)

with:

D: the specific absorbance;

m: the mass in g of the test part;

WMV: moisture content of the sample in %.

### 4.9. Statistical Analyses

The data were accessed as mean ± SEM. Statistical analysis was performed using one-way and two-way analysis of variance (ANOVA) using SigmaPlot 11.0 software (WPCubed GmbH, Munich, Germany). Post hoc tests were carried out using Tukey test for one-way or Duncan test for two-way ANOVA. The results were considered to be significant at *p* ≤ 0.05.

## 5. Conclusions

The present study proved that CSSE was safe up to 2000 mg/kg. It highlighted for the first time the analgesic potential of Moroccan saffron and displayed its dual effect (central and peripheral), unlike Iranian saffron. For the first time, this study sheds light on the possible physiological mechanism involved in the analgesic activity of saffron in general. This study implicated the activation of the opioidergic, cholinergic, dopaminergic, and adrenergic receptors, as well as the opening of ATP-sensitive K^+^-channels. The obtained data point to a multimodal mechanism of action: (i) an anti-inflammatory action and (ii) a modulation, through different physiological pathways, of the electric signal triggered by the nociceptors. Thus, the present study scientifically validates the traditional use of *C. sativus* stigma in the treatment of pain. However, further experimental studies focusing on the elucidation of the molecular mechanism behind the analgesic effect of CSSE are required; as well as clinical trials to obtain reliable data.

## Figures and Tables

**Table 1:** Effect of oral administration of a single dose of CSSE (2000 mg/kg, b.w) on the behavior and mortality of mice.

Group	Dose (mg/kg b.w p.o.)	Sex	D/T	Symptoms
Vehicle	0	Female	0/6	None
CSSE	2000	Female	0/6	None

CSSE: Hydro-Ethanolic Extract of Stigma of *C. sativus*; D/T: Number of deaths versus total number of mice.

**Table 2:** The relative organ weight of mice treated with a single oral dose of CSSE.

Organ	Relative Organ Weight (g/100 g b.w)	
	Control	CSSE (2000 mg/kg)
Heart	0.52 ± 0.04	0.59 ± 0.04
Lungs	0.93 ± 0.05	0.87 ± 0.08
Liver	0.06 ± 0.01	0.06 ± 0.01
Spleen	0.43 ± 0.06	0.51 ± 0.05
Right kidney	0.49 ± 0.01	0.54 ± 0.05
Left kidney	0.57 ± 0.01	0.56 ± 0.05
Stomach	0.77 ± 0.05	1.08 ± 0.16

CSSE: *C. sativus* stigma extract. The values are expressed as mean ± SEM (*n* = 6). No significant difference was recorded between CSSE-treated group and non-treated control (*p* < 0.05, ANOVA I Tukey’s test).

**Table 3:** Effect of a single oral dose of CSSE on blood hematological and biochemical parameters of mice.

Parameters		Unit	Control	CSSE (2000 mg/kg)
Hematological				
	White blood cell count (WBC)	10^3^/µL	6.8 ± 0.4	6.2 ± 0.8
	Red blood cell count (RBC)	10^6^/µL	8.8 ± 0.3	8.6 ± 0.4
	Hemoglobin (HGB)	g/dL	13.5 ± 0.8	13.2 ± 1.2
	Hematocrit (HCT)	%	44.4 ± 2.3	42.3 ± 3.6
	Platelets (PLT)	10^3^/µL	732 ± 40.3	694 ± 73.1
	Mean corpuscular volume (MCV)	fL	49.7 ± 1.3	47.4 ± 2.0
	Mean corpuscular hemoglobin (MCH)	pg	15.1 ± 0.7	16.0 ± 1.3
	Mean corpuscular hemoglobin concentration (MCHC)	g/dL	30.4 ± 2.1	31.7 ± 1.9
	Red cell distribution width (RDW)	%	22.0 ± 0.3	22.9 ± 0.7
	Mean platelet volume (MPV)	fL	8.0 ± 0.5	9.2 ± 0.8
	Neutrophil	%	10.6 ± 1.2	12.2 ± 2.2
	Lymphocyte	%	84.3 ± 5.9	76.9 ± 6.2
	Monocyte	%	4.7 ± 0.2	4.1 ± 0.5
Biochemical				
	Alanine aminotransferase (ALT)	UI/L	17 ± 2.00	16.25 ± 2.19
	Aspartate aminotransferase (AST)	UI/L	792 ± 60.50	838 ± 75.00
	Creatinine	mg/dL	3 ± 0.14	3 ± 0.09
	Urea	g/L	0.45 ± 0.04	0.50 ± 0.03
	Alkaline phosphatase (ALP)	UI/L	95.5 ± 7.5	89.75 ± 5.38
	Total bilirubin	mg/dL	0.30 ± 0.05	0.33 ± 0.09

CSSE: *C. sativus* stigma extract. The values are expressed as mean ± SEM (*n* = 6). No significant difference was recorded between CSSE-treated group and non-treated control (*p* < 0.05, ANOVA I Tukey’s test).

**Table 4:** Effect of CSSE on abdominal contractions induced by intraperitoneal injection of acetic acid in mice.

Group	Dose (mg/kg, b.w)	Number of Contortions	Inhibition of Pain (%)	Relative Activity Compared to Morphine (%)
Control	-	34.2 ± 1.5	-	-
Morphine	1	5.3 ± 0.7 ***	84.5 ± 0.5	100
CSSE	50	17.7 ± 0.3 ***	48.3 ± 0.8 ^+++^	57.2
	100	13.5 ± 0.4 ***	60.5 ± 1.0 ^+++^	71.6
	200	9.7 ± 0.4 ***	71.6 ± 0.9 ^++^	84.7

CSSE: Hydro-ethanolic extract of stigma of *C. sativus*; Morphine (1 mg/kg, i.p) is used as positive control. Each value represents the mean ± SEM (*n* = 6); *** Statistically significant (*p* ≤ 0.001) versus the non-treated control (ANOVA, Tukey’s test). ^++^ and ^+++^ Statistically significant (*p* ≤ 0.01) and (*p* ≤ 0.001), respectively versus morphine-treated group (ANOVA, Tukey’s test).

**Table 5:** Chemical characterization of Moroccan saffron according to saffron norm ISO 3632 2:2010 [17].

		Strength Indicator In:	A^1%^_1cm_ Measure	Saffron Samples	ISO 3632 Category 1	Quality Conformity
Pigments	Picrocrocin	Flavor	λ 257	525.5 ± 0.4	min. 70	First Quality Category
	Crocin	Coloring	λ 440	1126.3 ± 0.5	min. 200	First Quality Category
	Safranal	Aroma	λ 330	32.4 ± 0.2	min. 20; max. 50	First Quality Category
Moisture content		-	-	10.3 ± 0.24%	max 12%	Conform

## Data Availability

The data are available from the corresponding author on reasonable request.

## Sample Availability

Samples are available from the first author (M.A.T.).
